# Postoperative factors associated with hazardous alcohol use after metabolic and bariatric surgery

**DOI:** 10.1007/s00464-026-13270-x

**Published:** 2026-08-10

**Authors:** Lisa R. Miller-Matero, Daniel Saleh, Brittany Christopher, Maha Albujuq, Erin N. Haley, Alyssa Vanderziel, Jordan M. Braciszewski, Roland S. Moore, Maunda Snodgrass, Aaron Hamann, Arthur M. Carlin, Kristina M. Jackson

**Affiliations:** 1 Henry Ford Health, Behavioral Health, 1 Ford Place, 5E, Detroit, MI 48202, USA; 2 Henry Ford Health, Center for Health Policy and Health Services Research, Detroit, MI, USA; 3 College of Human Medicine, Michigan State University, East Lansing, MI, USA; 4 Pacific Institute for Research and Evaluation, Berkeley, CA, USA; 5 Department of Surgery, Henry Ford Health, Detroit, MI, USA; 6 Department of Psychiatry, Rutgers Robert Wood Johnson Medical School, New Brunswick, NJ, USA

**Keywords:** Metabolic and bariatric surgery, Alcohol use, Drinking motives, Psychiatric symptoms, Disordered eating

## Abstract

**Introduction:**

There is an increased risk of hazardous alcohol use following metabolic and bariatric surgery (MBS). Identifying risk factors of hazardous alcohol use, particularly factors that may differentiate risk among those with regular postoperative alcohol consumption, would inform postoperative monitoring.

**Method:**

Participants (*N* = 100) who were up to 5 years post-MBS and were consuming alcohol at least 2–3 times per month completed an assessment as part of a larger study. Measures assessed hazardous alcohol use, drinking motives, psychiatric symptoms, and disordered eating behaviors.

**Results:**

Participants were primarily female (87.0%), White (49.0%) or Black (44.0%), with a mean age of 48 years. Approximately 1 in 5 met the threshold for hazardous alcohol use. In bivariate analyses, clinically significant symptoms of depression (OR = 4.88, *p* = .01) and anxiety (OR = 8.21, *p* < .001) were associated with the presence of hazardous alcohol use. All four types of drinking motives were associated with greater likelihood of hazardous alcohol use: social (OR = 1.10, *p* = .03), coping (OR = 1.33, *p* < .001), enhancement (OR = 1.24, *p* < .001), and conformity (OR = 1.25, *p* = .01). Emotional eating scores and the presence of a food addiction were not significantly associated with hazardous alcohol use (*p* > .05). In a multivariable analysis, only the coping drinking motive remained statistically significant (OR = 1.26, *p* = .003).

**Conclusion:**

Drinking to cope with negative emotions may be a factor associated with increased risk of hazardous drinking among individuals who regularly consume alcohol after MBS. Interventions aimed at improving coping strategies could mitigate risk of hazardous alcohol use.

Metabolic and bariatric surgery (MBS) is the most effective and durable treatment to reduce excess weight and improve obesity-related medical conditions [1]. However, one concern for individuals after MBS is the risk for developing hazardous alcohol use. Following MBS, as many as one in five individuals may experience postoperative hazardous alcohol use or an alcohol use disorder [2]. Though the exact mechanisms of developing postoperative hazardous alcohol use are still being investigated, there are likely several contributing factors that place patients at higher risk. Suggested mechanisms include pharmacokinetic changes of alcohol as well as increases in the rewarding effects of alcohol following MBS [3].

Due to the increased risk for developing postoperative hazardous alcohol use, guidelines for the routine preoperative psychosocial evaluation suggest a careful assessment of current and past alcohol use history and preoperative management of high-risk individuals [4]. Some work has examined preoperative factors associated with postoperative alcohol use. Regular preoperative alcohol use (e.g., 2 + drinks per week) is one factor associated with greater risk of postoperative hazardous alcohol use [5]. Other preoperative factors associated with a greater likelihood of postoperative hazardous alcohol use include male sex, younger age, history of smoking, and limited social support [2, 5].

Although there is evidence that certain preoperative factors are associated with greater risk of postoperative hazardous alcohol use, these factors are applicable to a broad group of patients and, as such, are not very useful for identifying individuals at high risk for postoperative hazardous alcohol use. Therefore, it could be informative to understand whether there are more specific *postoperative factors* associated with increased risk. Regular postoperative alcohol use, relapse to smoking, and worsening psychiatric symptoms have been linked to greater risk of hazardous alcohol use [2]. Additional work examining other postoperative factors associated with increased risk could identify the potential targets for intervention. One area worthy of exploration is motives for alcohol use, as these are associated with drinking behaviors in other populations [6]. In addition, disordered eating behaviors, such as emotional eating and food addiction, are relatively common before MBS [7]. Some work has suggested that patients could change how they cope with emotions or experience “addiction transfer” from food to alcohol following MBS, yet most work has only looked at these variables preoperatively and findings are mixed [3]. As such, examining whether postoperative disordered eating behaviors (i.e., emotional eating and food addiction) are related to hazardous use would be useful. Moreover, not all individuals who engage in regular postoperative alcohol use develop a problem. Determining whether there are postoperative factors that distinguish risk for hazardous use among those who are engaging in postoperative alcohol use would further inform postoperative monitoring. The purpose of this study was to identify the postoperative factors associated with hazardous alcohol use among individuals who were engaging in regular alcohol use after MBS.

## Methods

### Participants and procedure

This study was a part of a larger project examining distal and proximal factors associated with alcohol use after MBS. Details of the study protocol have been previously reported [8]. This project was approved by the organization’s Institutional Review Board and all participants provided informed consent.

Participants who were between 6 months and 5 years post-MBS at a single health system were randomly selected and sent an email invitation to participate in a study examining postoperative alcohol use. If patients were interested, they were directed to complete a brief, Web-based eligibility screen. Patients were eligible if they reported consuming alcohol at least 2–3 times over the previous month. This inclusion criterion was selected as the goal of the project was to examine patients engaging in regular alcohol use. A research assistant contacted eligible patients to describe the study and obtain informed consent. Once consented, the participant completed the baseline assessment, which included the measures described below.

### Measures

#### Demographics.

Participants reported their age, sex, and race. Surgery type (Roux-en-Y gastric bypass or sleeve gastrectomy) was identified from medical records.

#### Hazardous alcohol use.

The alcohol use disorders identification test (AUDIT) was used to assess for hazardous alcohol use [9]. The AUDIT includes 10 items regarding the frequency and amount of alcohol use over the previous 6 months as well as consequences from alcohol use. Scores of 8 or greater suggest hazardous use. For analyses, the variable was dichotomized into hazardous use and non-hazardous use.

#### Preoperative alcohol use.

Chart reviews were conducted for all participants to review whether patients had a history of preoperative hazardous drinking. The AUDIT-C is routinely administered as a part of the preoperative psychosocial evaluation and scores from this measure were recorded [10]. Scores of 3 or greater for women and 4 or greater for men suggest hazardous alcohol use [10]. Because the AUDIT-C only assesses alcohol use in the past year, psychosocial evaluations from the medical records were reviewed to determine whether participants had a more remote history of hazardous drinking behaviors. This included self-reported binge drinking (i.e., 6 or more drinks in a sitting), a period of time with heavy drinking using the NIAAA definition (i.e., > 8 drinks per week for women or > 15 drinks per week for men) or history of alcohol use treatment. Participants were considered to have a history of preoperative hazardous alcohol use if they had an elevated AUDIT-C score and/or history of other markers of possible hazardous alcohol use.

#### Depression.

The Patient Health Questionnaire depression scale (PHQ-8) was used to determine the presence of clinically significant depressive symptoms [11]. Participants reported the frequency of depressive symptoms from “not at all” to “nearly every day.” Scores of 10 or greater indicated clinically significant depressive symptoms.

#### Anxiety.

The Generalized Anxiety Disorder-7 (GAD-7) [12] was used to determine the presence of clinically significant anxiety symptoms. Participants reported the frequency of anxiety symptoms from “not at all” to “nearly every day.” Scores of 10 or greater indicated clinically significant anxiety symptoms.

#### Emotional eating.

Emotional eating was measured with the Emotional Eating Scale (EES) [13]. Participants reported their urge to eat in response to 25 emotions that fall into 3 factors: anger/frustration, anxiety, and depression. Scores from items are combined for a composite score. Higher scores indicate greater levels of emotional eating. The EES was validated among individuals with obesity [13] and has been previously used with individuals pursuing MBS [14, 15].

#### Food addiction.

Though food addiction is not a recognized diagnosis in the DSM-V, the Yale Food Addiction Scale 2.0 (YFAS 2.0) assesses symptoms of food addiction that map onto the DSM-V criteria for substance use disorders [16]. In alignment with substance use disorder criteria, the YFAS 2.0 identifies up to 11 symptoms of food addiction. Participants were considered to have a food addiction if they had at least 3 symptoms and reported clinically significant distress and/or impairment on at least 1 of 2 items. The YFAS 2.0 has been validated among patients pursuing MBS [17].

#### Drinking motives.

The Drinking Motives Questionnaire-Revised (DMQ-R) [18] has 20 items across four subscales that measure different motives for alcohol use, which are social (i.e., to socialize; improve socialization), coping (i.e., to manage negative emotions), enhancement (i.e., to experience reward from use), and conformity (i.e., to fit in). Participants responded how frequently their drinking was motivated by each of the reasons listed in the past 6 weeks. Each subscale has five items with responses ranging from 1 (strongly disagree) to 4 (strongly agree). Items from each subscale are combined for a subscale score.

### Analyses

Analyses were conducted with SPSS version 29. Frequencies and descriptives were conducted to describe the study sample. Correlations were run to detect associations between psychiatric symptoms, drinking motives, and disordered eating behaviors. Bivariate logistic regressions were conducted to examine the associations between postoperative factors (depression, anxiety, disordered eating behaviors, and drinking motives) and past 6 month hazardous alcohol use. A multivariable logistic regression was conducted for factors that were significantly associated with hazardous alcohol use in the bivariate analyses.

## Results

There were 3,284 patients who were within the postoperative time frame of interest, of which about half (*n* = 1,618) were randomly selected and invited to complete the eligibility screen. Patients were selected from the overall list using a random number generator. There were 532 (32.9%) patients who completed the screen and of these, 191 (35.9%) were eligible. Research staff contacted participants in order of eligibility survey completion until the target number of 100 participants was enrolled (52.4% of those eligible), which was our a priori target based on the larger study. Participant characteristics are presented in Table 1. Participants were primarily female (87.0%), White (49.0%) or Black (44.0%), with a mean age of 48 years. The two types of procedures conducted were Roux-en-Y gastric bypass (55.0%) and sleeve gastrectomy (45.0%). Among this sample, 7.0% met the threshold for preoperative hazardous alcohol use and 19.0% met the threshold for current hazardous alcohol use. Two of the participants with preoperative hazardous alcohol use also reported scores consistent with current hazardous alcohol use; however, 17 of the participants (89.5%) did not have a history of preoperative hazardous alcohol use. There were 13.0% and 17.0% of participants who met the threshold for clinically elevated symptoms of depression and anxiety, respectively.

Correlations among psychiatric symptoms, disordered eating behaviors, and drinking motives are presented in Table 2. With the exception of the lack of significant association between emotional eating score and the conformity drinking motive, all other bivariate correlations were statistically significant.

In bivariate logistic regressions, clinically significant symptoms of depression and anxiety were associated with the presence of hazardous alcohol use (Table 3). Among individuals without hazardous alcohol use, 8.6% had significant depressive symptoms compared to 31.6% of those with hazardous alcohol use. There were 9.9% of individuals with significant anxiety among those without hazardous alcohol use compared to 47.4% of individuals with hazardous alcohol use. In addition, all types of drinking motives (social, coping, enhancement, and conformity) were associated with greater likelihood of hazardous alcohol use (Table 3). Emotional eating scores and the presence of a food addiction were not associated with likelihood of hazardous alcohol use (Table 3).

In a multivariable model that included all significant variables from the bivariate analyses, there was only one factor independently associated with likelihood of hazardous alcohol use (Table 4). Participants with greater levels of coping as a drinking motive had a greater likelihood of hazardous alcohol use.

## Discussion

The purpose of this study was to identify postoperative factors associated with hazardous alcohol use among individuals who were engaging in regular alcohol use after MBS. First, we found that most cases of postoperative hazardous drinking were new onset (89.5%), which is consistent with prior work suggesting increased risk of hazardous drinking after MBS [3].

In bivariate analyses, psychiatric symptoms (i.e., depression and anxiety) and drinking motives (social, coping, enhancement, and conformity) were all associated with greater likelihood of hazardous alcohol use. Disordered eating behaviors (e.g., emotional and food addiction) were not associated with the presence of hazardous alcohol use. In the multivariable model, only the coping drinking motive continued to be independently associated with hazardous alcohol use. In other samples, both coping and enhancement were strongly associated with drinking problems [6]. This study suggested that following MBS, drinking to cope may be a stronger predictor of hazardous alcohol use than drinking for enhancement. This was surprising given that individuals are shown to experience increased reward from postoperative alcohol use, which has been a hypothesized mechanism of postoperative problematic alcohol use [3, 19]. Findings from the current study supported qualitative work with a small sample (*N* = 20) where participants reported that coping with negative mood was a driver of alcohol use following MBS [20, 21]. The current study extended this work and suggested that drinking to cope may be associated with hazardous use. Although there is little quantitative research examining drinking motives after MBS, one previous study examined drinking motives prior to and 6-months post-MBS [22]. In this study, there was a small group of individuals who increased alcohol use 6 months after MBS. Within this group, scores on the coping subscale also increased (with no significant changes in the other drinking motives), suggesting that there may be a relationship between drinking to cope and increased alcohol use 6-months after MBS. The current study expanded upon these findings to include individuals more remote from surgery (up to five years) and also examined hazardous alcohol use as the outcome. Taken together, drinking to cope may start soon after surgery and result in increased alcohol use, which could then lead to hazardous alcohol use.

There were several variables that were statistically significant in bivariate analyses that were no longer significant in the multivariable model, including elevated depression and anxiety scores and the other types of drinking motives (e.g., social, enhancement, and conformity). This may have occurred due to multicollinearity, given the significant correlations among these variables. Thus, this does not mean that these factors are not associated with hazardous alcohol use and that there could be a clinically meaningful association. In other patient samples, there is a strong link between psychiatric symptoms (i.e., depression and anxiety) and alcohol use disorders [23], so it is not be surprising if this pattern emerged after MBS as well. As such, it may still be important to assess symptoms of depression and anxiety and all types of drinking motives in postoperative monitoring.

There were several strengths of this study. Few studies have examined drinking motives after MBS, and understanding motives for drinking has important implications for postoperative monitoring and interventions. In addition, a novel aspect of this work was that the sample included all individuals who were regularly drinking and could therefore examine the differences between those with and without hazardous alcohol use. However, it is important to note some limitations of this study. First, the timing of some measurements differs. For example, symptoms of depression and anxiety query symptoms over the previous two weeks whereas hazardous alcohol use is over the past six months. Second, it is possible that some individuals who did not meet the threshold for hazardous use at the time of the study could develop hazardous use in future. Longitudinal follow-up to determine whether associations are maintained would be useful. As mentioned previously, the study had a smaller sample size and may not have been adequately powered for some variables. Finally, given that only about a third of patients completed the eligibility survey, there could be a selection bias in those who volunteered to participate.

In conclusion, in this study of individuals who engaged in regular postoperative alcohol use, coping as a drinking motive was the only variable that was independently associated with the presence of hazardous alcohol use. Findings suggest that individuals who consume alcohol to cope with negative emotions may be at greater risk of developing postoperative hazardous alcohol use compared to individuals who are using alcohol but not for the purposes of coping with negative mood. In postoperative monitoring, clinicians may want to ask about motives for drinking and determine if coping is a precursor of drinking behavior. In addition, interventions that focus on developing adaptive coping strategies may be useful for reducing alcohol use. Future research may want to test whether improving adaptive coping strategies could mitigate the risk of hazardous alcohol use.

## Figures and Tables

**Table 1:** Participant characteristics, *N* = 100

	M	SD

Days since undergoing MBS	1169.67	394.62
Age (yrs)	48.12	10.35
Emotional eating score	46.97	16.95
Drinking motive—social	12.62	5.79
Drinking motive—coping	10.16	6.04
Drinking motive—enhancement	10.75	5.36
Drinking motive—conformity	6.39	3.22

	%	*n*

Gender		
Female	87.0	87
Male	13.0	13
Race		
White	49.0	49
Black/African American	44.0	44
Other/Multiracial	7.0	7
Surgery type		
Roux-en-Y gastric bypass	55.0	55
Sleeve gastrectomy	45.0	45
Preoperative hazardous alcohol use	7.0	7
Current hazardous alcohol use	19.0	19
Significant depressive symptoms	13.0	13
Significant anxiety symptoms	17.0	17
Food addiction	11.0	11

**Table 2:** Correlations among postoperative psychiatric symptoms, disordered eating behaviors, and drinking motives

	1	2	3	4	5	6	7	8
1. Depressive symptoms	–							
2. Anxiety symptoms	.86**	–						
3. Emotional eating score	.38**	.30**	–					
4. Food addiction symptoms	.35**	.26*	.27**	–				
5. Drinking motive—social	.32**	.34**	.24*	.25*	–			
6. Drinking motive—coping	.48**	.60**	.30**	.32**	.39**	–		
7. Drinking motive—enhancement	.24**	.29**	.29**	.28**	.72**	.66**	–	
8. Drinking motive—conformity	.38**	.48**	.13	.40**	.48**	.46**	.44**	–

* *p* < .05

** *p* < .001

**Table 3:** Bivariate associations of psychiatric symptoms, disordered eating, and drinking motives with hazardous alcohol use

	No hazardous alcohol use		Had hazardous alcohol use		B	SE	OR (95% CI)	*p*
	M	SD	M	SD				

Emotional eating score	45.54	16.30	53.05	18.76	.03	.02	1.03 (.996, 1.06)	.09
Drinking motive—social	12.01	5.32	15.21	7.04	.09	.04	1.10 (1.01, 1.20)	.03
Drinking motive—coping	8.26	3.93	18.26	6.84	.28	.06	1.33 (1.18, 1.49)	< .001
Drinking motive—enhancement	9.53	4.25	15.95	6.52	.21	.05	1.24 (1.12, 1.37)	< .001
Drinking motive—conformity	5.86	1.77	8.63	6.05	.22	.08	1.25 (1.06, 1.47)	.01

	%	*n*	%	*n*	B	SE	OR (95% CI)	*p*

Significant depressive symptoms	8.6	7	31.6	6	1.59	.63	4.88 (1.41, 16.85)	.01
Significant anxiety symptoms	9.9	8	47.4	9	2.11	.59	8.21 (2.58, 26.18)	< .001
Food addiction	8.6	7	21.1	4	1.04	.69	2.82 (.73, 10.85)	.13

**Table 4:** Multivariable analysis of factors associated with hazardous alcohol use

	B	SE	aOR (95% CI)	*p*
Drinking motive—social	−.15	.11	.86 (.69, 1.07)	.17
Drinking motive—coping	.23	.08	1.26 (1.08, 1.47)	.003
Drinking motive—enhancement	.17	.12	1.18 (.94, 1.48)	.15
Drinking motive—conformity	.11	.15	1.12 (.84, 1.49)	.43
Significant depressive symptoms	.23	1.20	1.25 (.12, 13.28)	.85
Significant anxiety symptoms	.04	1.22	1.04 (.10, 11.47)	.97
